# Kinesins Modify ERR1-Dependent Transcription Using a Conserved Nuclear Receptor Box Motif

**DOI:** 10.3390/ijms24043795

**Published:** 2023-02-14

**Authors:** A. M. Pramodh Bandara Seneviratne, Sarah Lidagoster, Sofia Valbuena-Castor, Kareena Lashley, Sumit Saha, Aleksandra Alimova, Geri Kreitzer

**Affiliations:** 1 CUNY School of Medicine, City College of New York, New York, NY 10031, USA; 2 Department of Molecular, Cellular and Biomedical Sciences, CUNY School of Medicine, City College of New York, New York, NY 10031, USA

**Keywords:** kinesin, estrogen related receptor alpha, transcriptional regulation, LxxLL

## Abstract

Kinesin family motors are microtubule (MT)-stimulated ATPases known best as transporters of cellular cargoes through the cytoplasm, regulators of MT dynamics, organizers of the mitotic spindle, and for insuring equal division of DNA during mitosis. Several kinesins have also been shown to regulate transcription by interacting with transcriptional cofactors and regulators, nuclear receptors, or with specific promotor elements on DNA. We previously showed that an LxxLL nuclear receptor box motif in the kinesin-2 family motor KIF17 mediates binding to the orphan nuclear receptor estrogen related receptor alpha (ERR1) and is responsible for the suppression of ERR1-dependent transcription by KIF17. Analysis of all kinesin family proteins revealed that multiple kinesins contain this LxxLL motif, raising the question as to whether additional kinesin motors contribute to the regulation of ERR1. In this study, we interrogate the effects of multiple kinesins with LxxLL motifs on ERR1-mediated transcription. We demonstrate that the kinesin-3 family motor KIF1B contains two LxxLL motifs, one of which binds to ERR1. In addition, we show that expression of a KIF1B fragment containing this LxxLL motif inhibits ERR1-dependent transcription by regulating nuclear entry of ERR1. We also provide evidence that the effects of expressing the KIF1B-LxxLL fragment on ERR1 activity are mediated by a mechanism distinct from that of KIF17. Since LxxLL domains are found in many kinesins, our data suggest an expanded role for kinesins in nuclear receptor mediated transcriptional regulation.

## 1. Introduction

Kinesin family motors are microtubule stimulated ATPases that transport a large variety of cellular cargo along microtubules, regulate microtubule dynamics, and ensure the equal segregation of genetic material during cell division [1]. In the human genome, there are 45 distinct kinesin genes. Apart from their canonical functions, a growing body of evidence highlights a non-canonical role for kinesins in the regulation of transcription. Members of the kinesin-2 (KIF17) and kinesin-4 (KIF4, KIF7, Costal-2, BC12) families have well-documented roles in diverse species and across phyla in modifying the functions of transcription regulators or binding directly to specific promotor elements in DNA.

Mechanistically, the participatory role of kinesins in the regulation of transcription varies greatly. The kinesin KIF7 (Costal-2 in flies) sequesters Gli proteins (cubitis interruptus in flies) in the tip compartment of primary cilia (or cytoplasm in flies) and regulates their post-translational processing and transcriptional function in hedgehog signaling [2,3,4,5,6,7,8]. The interaction of KIF7 with Gli proteins is mediated by a coiled-coil domain in KIF7 that mimics DNA and binds to zinc fingers in Gli [9]. KIF4 sequesters poly-ADP-ribosyltransferase (PARP-1), a DNA repair protein and transcriptional regulator, in the nucleoplasm and inhibits its enzymatic activity until calcium influx is stimulated by membrane depolarization, resulting in CamKII-dependent release of PARP-1 from KIF4 [10]. The interaction of KIF4 with PARP-1 is mediated by the globular kinesin tail domain. Overexpression of a dominant-negative KIF4 tail domain in mouse neurons resulted in increased cell death, consistent with a role for PARP-1 in regulating anti-apoptotic gene expression. Kin4A in *O. sativa japonica* (also known as BC12, BC2, or GDD1) contains a bZIP motif and binds directly to the kaurene oxidase promotor. Kin4A-induced activation of *ent*-kaurene oxidase expression is required for the biosynthesis of gibberellic acid and cell elongation. Disruption of Kin4A binding to the kaurene oxidase promotor results in plant dwarfism [11]. The testis specific mouse KIF17b regulates the cytoplasmic-nuclear shuttling of the activator of CREM mediated transcription (ACT) and CREM activity [12]. The interaction between KIF17b and ACT is mediated by an ~100 amino acid region within the KIF17b stalk domain [13]. In a complex, ACT-KIF17b translocates from the cytoplasm to the nucleus by a cAMP- and PKA-dependent but microtubule-independent mechanism.

We previously showed that the human kinesin 2 family member KIF17 interacts with the orphan nuclear receptor estrogen related receptor alpha (ERR1) [14]. Binding between KIF17 and ERR1 was mapped to the C-terminal tail domain of KIF17 and the C-terminal half of ERR1, which contains the ligand binding and activation factor 2 (AF2) domains [14]. The interaction of KIF17 with ERR1 is reduced significantly by deletion of 12 amino acids at the beginning of the KIF17 tail domain that includes an LxxLL nuclear receptor (NR) box. The LxxLL motif is conserved in transcriptional cofactors that bind nuclear receptors at AF2 domains [15] and is a multifunctional binding sequence involved in transcriptional regulation [16]. Thus, KIF17 appears to engage ERR1 in a manner similar to other regulatory cofactors. Consistent with this idea, expression of the KIF17 NR box in breast cancer cells inhibited the expression of a subset of ERR1 gene targets, suggesting that KIF17 is a selective, competitive inhibitor of ERR1 cofactor binding in the nuclear compartment. 

ERR1 controls the expression of genes involved in adaptive energy metabolism, cell proliferation, cell migration, cell differentiation, cell survival, and autophagy, among others [17,18,19,20,21]. As an orphan nuclear receptor, ERR1 is thought to be constitutively active. However, expression of its many gene targets is controlled, at least in part, by regulated binding to different transcriptional cofactors [22,23,24,25,26,27]. With the discovery of cholesterol as a ligand for ERR1 [28,29], ERR1 could potentially be reclassified as an adoptive orphan. Because ERR1 expression and activity are also correlated with poor prognosis in many cancers [30,31,32], ERR1 and identification of its potential modulators is the subject of active research. 

To determine if the mechanism used by KIF17 to regulate ERR1-dependent transcription is unique, we identified additional kinesin family members containing LxxLL motifs and assessed the effect of manipulating these kinesins on ERR1-mediated transcription. Here, we show that an LxxLL domain present in the kinesin-3 family motor KIF1B interacts with and regulates ERR1-mediated transcription in breast cancer cells. This regulation leads to global suppression of ERR1 transcriptional targets, distinguishing it from the selective effects of KIF17 on ERR1 gene targets, and provides additional insight into the role of kinesins and the LxxLL motif in transcriptional regulation.

## 2. Results

### 2.1. Identification of Kinesin Family Motor Transcriptional Regulator Candidates

**Figure 1:**
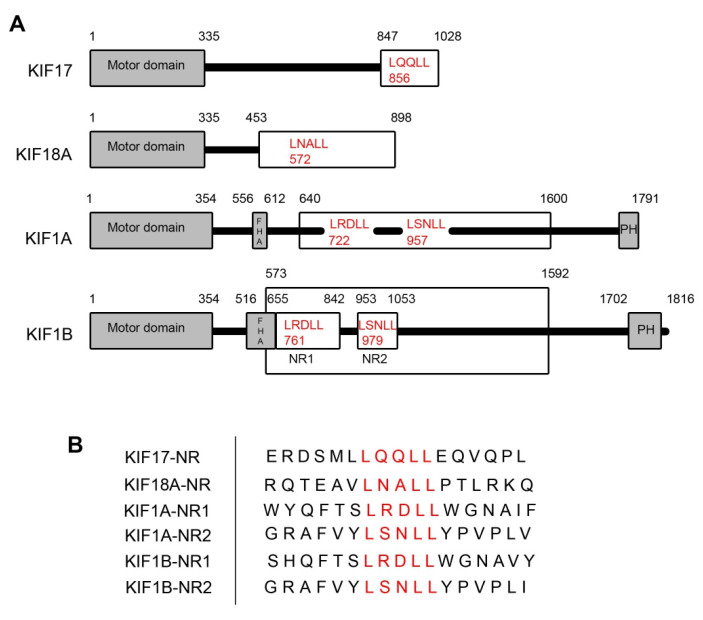
Kinesin family motors containing LxxLL nuclear receptor box motifs. (**A**) Schematic diagrams showing the kinesins with LxxLL motifs used in this study. The kinesin motor domains are indicated by the grey boxes. Kinesin fragments used to generate expression plasmids are shown in the white boxes. LxxLL motifs within these fragments are shown in red text, with numbers indicating the position of the first leucine of the motif. The numbers above each diagram represent the amino acid positions within the full-length kinesin sequence. (**B**) Sequence alignment of LxxLL domains of the kinesin constructs used in the study. Red lettering represents the LxxLL motifs.

To assess if kinesin family motors other than KIF17 have the potential to act as transcriptional regulators of ERR1 in the nucleus, we searched the sequences of all kinesin family motors in the human genome to identify those which contain nuclear receptor box (NR box) LxxLL motifs (Table 1). We then selected candidates for experimental assessment based on (i) the location of the LxxLL motif within the protein and (ii) the presence and predicted strength of nuclear localization signals (NLS). We eliminated kinesins that contained LxxLL motifs solely in the microtubule-binding motor domain, within or adjacent to coiled-coil domains, and within other well-characterized interaction domains (e.g., forkhead homology [FHA], plekstrin homology [PH], or phox homology [PX] domains). We also eliminated kinesins that lacked at least one predicted NLS outside the motor domain. The remaining kinesins were then sorted by the predicted strength of the NLS using NLS mapper (https://nls-mapper.iab.keio.ac.jp/cgi-bin/NLS_Mapper_form.cgi), where only kinesins with NLS sequences predicted to result in strong nuclear localization, or localization to both the cytoplasm and nucleus [33,34], were considered further. Kinesins that did not contain a classical monopartite NLS [35] were also excluded, although some may be appropriate targets for future study. Finally, kinesins without reported functions in interphase and kinesins that act as MT depolymerases were also eliminated. Using these constraints, only KIF17, KIF18A, KIF1A, and KIF1B met the criteria for inclusion. KIF17 and KIF18A each contain one LxxLL motif, while KIF1A and KIF1B each contain two LxxLL motifs (denoted KIF1A-NR1, KIF1A-NR2, KIF1BNR1, and KIF1B-NR2) (Figure 1).

### 2.2. KIF1B-NR1 Inhibits ERR1-Dependent Transcription

**Figure 2:**
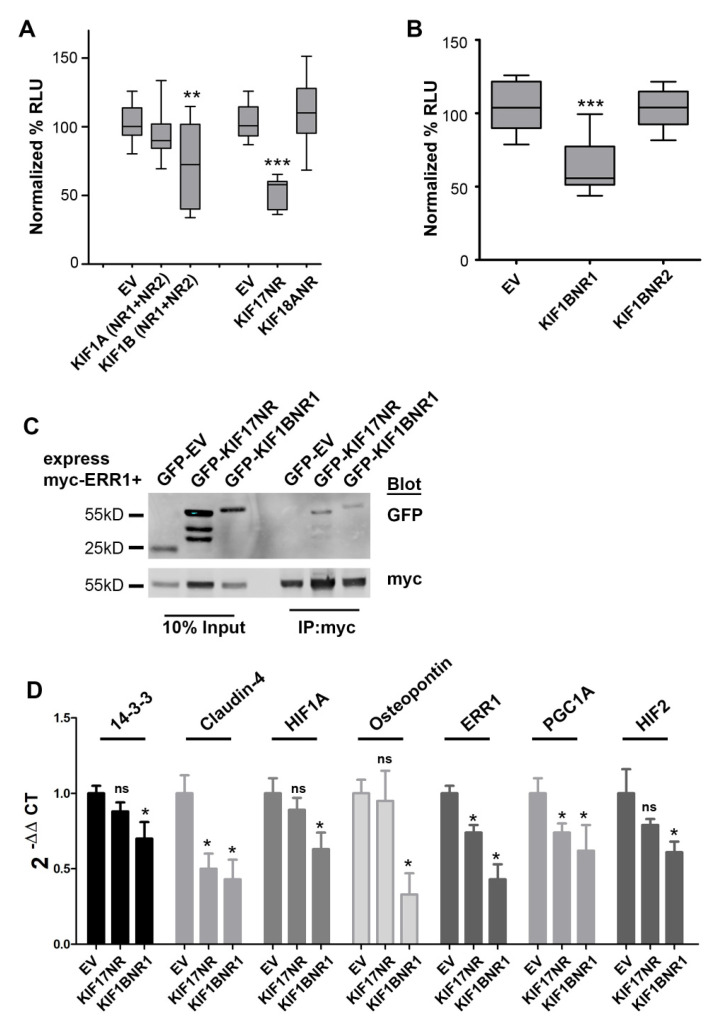
KIF1BNR1 binds to and inhibits ERR1 transcriptional activity. (**A**) Luciferase reporter assay in MCF7 cells co-expressing ERRE-Luc with GFP-tagged empty vector (EV), KIF1A fragment containing nuclear receptor box motifs 1 and 2 (KIF1A-NR1+NR2), KIF1B fragment containing both nuclear receptor box motifs 1 and 2 (KIF1B-NR1+NR2), KIF17 nuclear receptor box motif (KIF17-NR) or KIF18A nuclear receptor box motif (KIF18A-NR). All graphs show luminescence values normalized to GFP-EV. Data are pooled from ≥3 experiments performed in triplicate. Error bars = SEM, ** *p* > 0.05, *** *p* < 0.05. (**B**) Luciferase reporter assay in MCF7 cells co-expressing ERRE-Luc with GFP-tagged empty vector (EV), KIF1B nuclear receptor box motif 1 (KIF1B-NR1) or nuclear receptor box motif 2 (KIF1B-NR2). All graphs show luminescence values normalized to GFP-EV. Data are pooled from ≥5 experiments performed in triplicate. Error bars = SEM, *** *p* < 0.05. (**C**) Co-immunoprecipitation analysis of GFP-EV, GFP-KIF17-NR, and GFP-KIF1B-NR1 with myc-ERR1 in HEK-293T cells. Inputs represents 10% of total lysates. (**D**) qPCR analysis of known ERR1 targets in MCF7 cells. Error = SEM. * *p* < 0.05, not significant (ns).

We generated epitope or fluorescently tagged expression plasmids encoding fragments of KIF1A, KIF1B, and KIF18A that contained the LxxLL motifs identified in these kinesins (denoted *tag*-KIF*X*-NR). KIF17 constructs containing the LxxLL were described previously [14]. For KIF1A and KIF1B, we first generated constructs that contained both of the LxxLL motifs residing outside the motor domain. We next co-expressed these kinesin fragments in MCF7 breast cancer cells with a plasmid containing the ERR1 responsive element fused to luciferase (ERRE-Luc) and performed luminescence reporter assays to determine if expression of the LxxLL regions of these kinesins alters ERR1-dependent transcription. As a negative control, we expressed either myc- or GFP-empty vector (denoted *tag*-EV). Expression of GFP-KIF17-NR reduced luciferase production by 49%, consistent with our prior study [14]. Expression of GFP-KIF1B-NR1+NR2 decreased luciferase production by 28%, but with significant variability. By contrast, expression of GFP-KIF1A-NR1+NR2 or KIF18A-NR had no significant impact on luciferase production from the ERRE-Luc reporter (Figure 2A). The significant variation in luciferase production observed in cells expressing GFP-KIF1B-NR1+NR2 prompted us to create two additional expression plasmids encoding single NR box regions of KIF1B, KIF1B-NR1, and KIF1B-NR2 (Figure 1B). Expression of GFP-KIF1B-NR1 in MCF7 cells inhibited luciferase production by ERRE-Luc by 40% compared with empty vector control, while KIF1B-NR2 had no significant effect (Figure 2B). Collectively, these data suggest that KIF1B, through the NR1-containing region, may be another regulator of ERR1-mediated transcription.

### 2.3. KIF1B-NR1 Interacts with ERR1

We performed co-immunoprecipitation analysis to determine if the effects of KIF1B-NR1 on ERR1-dependent transcription results from an interaction of these proteins. We co-expressed myc-ERR1 and GFP-KIF1B-NR1 in HEK293T cells. As a positive control for the interaction of kinesin with ERR1, we co-expressed myc-ERR1 with GFP-KIF17-NR in HEK293T cells. One day after transfection, we lysed the cells, reserved 10% of this total lysate, and incubated the remainder of cell lysates with anti-myc antibody to immunoprecipitate myc-ERR1. In these experiments, we found that both GFP-KIF17-NR and GFP-KIF1B-NR1 co-immunoprecipitated with myc-ERR1 (Figure 2C). From these data, we conclude that the inhibitory effect of expressing KIF1B-NR1 on ERR1-dependent transcription is likely mediated by the binding of KIF1B-NR1 to ERR1. Thus, while KIF1B contains two LxxLL NR box motifs, only NR1 region is involved in modifying ERR1-dependent transcription. 

### 2.4. KIF1B-NR1 Globally Inhibits ERR1 Target Genes

We showed previously that expression of the KIF17-NR inhibits transcription of a subset of reported ERR1-gene targets [14]. Since expression of either KIF1B-NR1 or KIF17-NR inhibits ERR1-dependent transcription in reporter assays, we next tested if the expression of KIF1B-NR1 inhibits the same or different gene targets of ERR1 using qPCR. For these experiments, we generated MCF7 stable cell lines expressing either GFP-EV as a negative control, GFP-KIF1B-NR1, or GFP-KIF17-NR. We then compared the effects KIF1B-NR1 with that of KIF17-NR and empty vector on the expression of seven genes under the control of ERR1. Expression of KIF1B-NR1 significantly inhibited mRNA expression of all seven gene targets tested (Figure 2D). By contrast, and consistent with our prior studies in MDA-MB-231 breast cancer cells [14], expression of KIF17-NR selectively inhibited expression of ERR1 targets, including ERR1, PGC1A, and claudin-4, but had no significant effect on the mRNA levels of 14-3-3ζ, HIF1A, HIF2, and osteopontin. These data demonstrate that although both KIF1B-NR1 and KIF17-NR bind ERR1 and inhibit its transcriptional activity in reporter assays, they do not likely act by the same mechanisms since their effects on the transcription of specific ERR1-dependent gene targets are distinct. 

### 2.5. KIF1B-NR1 Inhibits Nuclear Import of ERR1 by Sequestering ERR1 in the Cytoplasm

**Figure 3:**
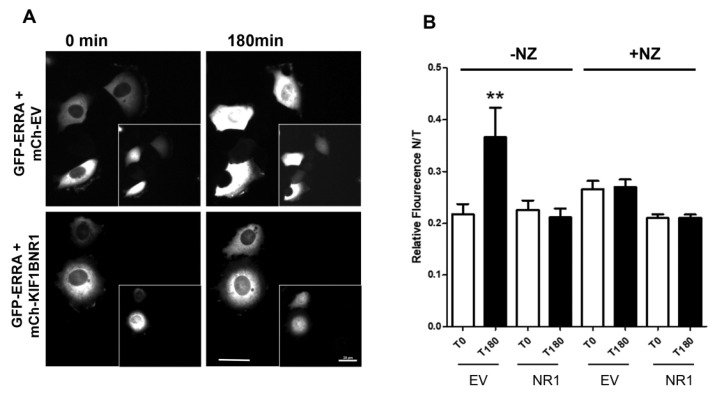
Nuclear accumulation of newly expressed ERR1 is inhibited by expression of KIF1BNR1 and is independent of an intact microtubule network. (**A**) MCF-7 cells injected with GFP-ERR1 and control, mCh-EV, or mCh-KIF1B-NR1 cDNAs. Images show GFP-ERR1 in first and last frames of time-lapse recordings. Insets: mCh-EV control and mCh-KIF17-NR in injected cells. Scale bars = 20 μm. (**B**) Graphs showing the fraction of nuclear GFP-ERR1 as a percentage of total cellular GFP-ERR1 (N/T fluorescence) in cells co-expressing mCh-EV or mCh-KIF1B-NR1. GFP-ERR1 fluorescence intensities (nuclear and total) were quantified at the first and last time points in recordings of cells with intact microtubules (-NZ), and in cells where microtubules are disrupted with nocodazole (+NZ). Error bars = SEM. ** *p* < 0.05.

The non-selective inhibition of ERR1 target gene expression by KIF1B-NR1 suggests that it may act as a dominant-negative inhibitor that sequesters ERR1 from DNA. To test this idea, we used time-lapse fluorescence microscopy to monitor the subcellular distribution of newly synthesized ERR1 in the presence of empty vector control or KIF1B-NR. We co-expressed mCh-ERR1 and GFP-EV or GFP-KIF1B-NR1 in MCF-7 cells by intra-nuclear injection of plasmid DNA. Ninety minutes after microinjection, the protein products of these cDNAs could be detected microscopically. At this time, cells were transferred to a temperature-controlled chamber on the microscope and the distribution of mCh-ERR1 and GFP-EV or GFP-KIF1B-NR1 was monitored at 10 min intervals for 3 h at 35 °C (Figure 3A). From these images, we quantified the cytoplasmic and nuclear ERR1 fluorescence over time to determine the impact of expressing KIF1B-NR1 on translocation of newly synthesized mCh-ERR1 from the cytoplasm to the nucleus (Figure 3B). We routinely measured ~20–30% of the total mCh-ERR1 in the nucleus at the start of all recordings. In control cells expressing GFP-EV and mCh-ERR1, the fraction of nuclear mCh-ERR1 increased by ≥two-fold over the course of 3 h. Conversely, in cells expressing mCh-ERR1 and GFP-KIF1B-NR1, the translocation of mCh-ERR1 from the cytoplasm to the nucleus was inhibited nearly completely. As expected, expression of GFP-KIF1B-NR2, which does not bind ERR1, had no effect on nuclear translocation of co-expressed mCh-ERR1 in these assays.

To determine if the effects of KIF1B-NR1 on the nuclear import of ERR1 are dependent on an intact MT network, we treated cells with nocodazole to depolymerize MTs. Nocodazole was added to cells immediately after cDNA injection, and cells were continuously incubated in the drug during the 3 h time-lapse recording. Treatment with nocodazole (NZ) inhibited entry of newly synthesized ERR1 into the nucleus in cells expressing mCh-ERR1 and either GFP-EV or GFP-KIF1B-NR1. This inhibition of ERR1 translocation into the nucleus by MT disruption in estrogen receptor (ER)-positive MCF7 cells differs from what we observed previously in experiments using ER-negative MCF10A and MDA-MB-231 cells. In ER-negative cells, newly synthesized ERR1 accumulates in the nucleus in the absence of MTs [14]. Considered together, these findings suggest that the nuclear translocation of ERR1 may be influenced by its ability to dimerize with ER [36], and are consistent with data showing that nuclear translocation of ER is MT- and MT motor-dependent [37,38,39]. The inhibitory effect of MT disrupting agents on nuclear entry of ERR1 in ER-positive cells, but not in ER-negative cells, indicates that ERR1 is regulated differently in ER-negative and ER-positive cell types.

## 3. Discussion

Many kinesin family motors encode one or more LxxLL motifs, a sequence that is conserved in nuclear receptor coactivator proteins and is known as the nuclear receptor box motif. The function of this motif in kinesins, however, is not known. Further, although roles for several kinesins in transcription regulation have now been documented, the roles of kinesin LxxLL motifs in this process have gone largely unexplored. We showed previously that the LxxLL motif contained within the tail domain of KIF17 is both necessary and sufficient to modify expression of ERR1-dependent genes in breast cancer cells [14]. In our prior work, we also demonstrated that KIF17 regulates ERR1 function in a manner which suggests it competes with nuclear receptor transcriptional co-activators in the nucleus. The goal of this study was to assess if LxxLL motifs encoded by additional kinesin family motors are also able to engage ERR1 and modify its activity. 

The presence of one or more LxxLL motifs in numerous kinesin family members made it necessary to prioritize the kinesin motors to be examined using stringent criteria. First, since the microtubule and ATP-binding domains of all kinesins are highly conserved [40] and are required to execute the motor functions of these proteins, we hypothesized that only LxxLL domains outside the kinesin motor domain would be functionally relevant in engaging transcription regulators. LxxLL motifs located within or adjacent to the kinesin neck-linker, neck-coil, coiled-coils, and other well-characterized interaction domains were also eliminated from consideration. Second, because nuclear receptor function and transcriptional regulation occurs in the nucleus, we limited our search to kinesins encoding a strong NLS outside the kinesin motor domain. Non-classical NLS [35] were also eliminated, but should be considered further in future studies. For example, KIF11, a kinesin involved in the separation of spindle poles during mitosis [41], was shown to interact with thyroid hormone receptor beta in yeast-2-hybrid assays, and with NCoR1 in affinity-MS proteomic assays [42]. Although the functional significance of these interactions is not yet known, KIF11 possesses an LxxLL motif outside of its motor domain, but it does not possess a classical NLS and, instead, encodes a non-classical, long linker (20 amino acid), bipartite NLS of moderate strength. As there is no evidence to date indicating KIF11 localizes to the nucleus in interphase cells, we chose to eliminate it as a candidate in our study. However, a broader consideration of kinesin LxxLL domains, along with rigorous examination of kinesin localization to the nucleus in interphase, could justify further investigation of KIF11 and other motors with respect to transcriptional regulation. 

The kinesin family motors that conformed best to our criteria included KIF17, KIF18A, KIF1A, and KIF1B. Consistent with our prior work, expression of the LxxLL contained in KIF17-NR inhibited ERR1-dependent transcription. Of the other kinesins tested, ERR1-dependent transcription was affected only by the expression of KIF1B-NR1. Of note, the core LxxLL motifs in KIF1B-NR1 (LRDLL) and KIF1B-NR2 (LSNLL) differ, and are also flanked by distinct amino acids. These factors could limit the availability of KIF1B-NR2 for interaction with ERR1, as compared with KIF1B-NR1, and may account for the observed differences in the impact of expressing KIF1B-NR1 and KIF1B-NR2 on the function of ERR1, as reported previously for multiple nuclear receptors [43,44,45,46,47]. At present there is no structure available for either full length KIF1B or for the relevant regions of KIF1B examined here. However, predictive structures of KIF1B-NR1 and KIF1B-NR2 regions in AlphaFold [48] (https://alphafold.ebi.ac.uk/entry/O60333), and analysis of solvent accessible surface area (ChimeraX, https://www.cgl.ucsf.edu/chimerax/), indicate that the KIF1B-NR1 is more exposed than that of KIF1B-NR2. Further, while the LxxLL motifs of KIF1A-NR1 and KIF1B-NR1 (LRDLL) are identical, the amino acid sequences (starting at −3 from the first leucine, and +3 from the last leucine in the core motif) flanking the LxxLL in KIF1A-NR1 and KIF1B-NR1 differ. This raises the possibility that amino acids located further upstream and downstream of the core LxxLL motif may contribute to the ability of kinesins to bind and regulate ERR1, as described previously for the binding of transcriptional co-activators to estrogen receptors and thyroid hormone receptors [44]. Thus, the amino acids flanking LxxLL core motifs and the conformation of these regions may contribute to the ability of NR boxes to bind and exert effects on ERR1. This possibility will need to be addressed further in future work.

The kinesin-8 family motor KIF18A also contains LxxLL motifs, and was reported to bind estrogen receptors in the cytoplasm of stromal cells in the presence of estradiol [49]. However, the interaction between KIF18A and estrogen receptors was not mapped, and thus the significance of the LxxLL motif to KIF18A and estrogen receptor binding is not known. Furthermore, the effect of KIF18A on estrogen receptor-dependent transcription was not reported, so it is unclear if KIF18A acts to modify estrogen receptor-mediated gene expression. In our assays, expression of KIF18A fragments containing the LxxLL domain had no impact on ERR1-mediated transcription. As such, it is not yet clear whether KIF18A LxxLL motifs are functionally significant.

Our prior work and the data shown here suggest at least two possible mechanisms by which the interaction of a kinesin with ERR1 regulates ERR1 function; one possible mechanism is dominated by cytoplasmic regulation and the other by regulatory control in the nucleus. KIF1B regulates ERR1-dependent transcription globally by sequestering ERR1 in the cytoplasm. By comparison, KIF17 regulates ERR1-dependent transcription selectively, by modifying ERR1 function in the nucleus. Global inhibition of ERR1-dependent transcription by expressed KIF1B-NR1 would be expected to broadly impact cellular processes regulated by ERR1-dependent genes, while the selective inhibition of ERR1-dependent genes by KIF17 would likely affect only a subset of cellular processes regulated by ERR1 actions. Further studies are needed to test this directly and to identify the specific ERR1-dependent cellular behaviors that are modulated by KIF17 and KIF1B. Interestingly, the subset of ERR1-dependent genes affected by the expression of KIF17-NR in MCF-7 differed from those affected in MDA-MB-231 cells [14]. This variance could be due to the differential activation of gene expression by ERR1 in the presence of ER, mediated by ERR1-ER heterodimers in ER-positive cells such as MCF-7 [36,50,51]. MDA-MB-231 are triple-negative breast cancer cells, and thus do not express ER. The selective effects of expressing KIF17-NR on ERR1-dependent transcription could also be due to the displacement of a subset of ERR1 transcriptional coactivators that may be differentially expressed in ER-positive and ER-negative cells. 

In summary, the work presented here, along with prior studies documenting the role of kinesin motors in regulating gene transcription, highlight gaps in our knowledge of non-canonical kinesin functions. However, it is clear that kinesins use multiple mechanisms to exert effects on the activity of transcription factors. Further investigation will be needed to determine if additional nuclear receptors are regulated by kinesins, and if LxxLL motifs in additional kinesin family motors are utilized to regulate gene expression more broadly. If so, LxxLL sequences could be envisioned as tools to manipulate the expression of ERR1 gene targets pharmacologically and selectively. 

## 4. Materials and Methods

### 4.1. Cell Culture, Transfection, and Microinjection

MCF7, MDA-MB-231, and HEK293T cells (ATCC) were cultured in DMEM (Corning, 4.5 g/liter glucose, with L-glutamine and sodium pyruvate, pH 7.2) with 5% fetal bovine serum (FBS #S11150, Lot #F18071, Atlanta Biologicals/R&D Systems, Flowery, GA, USA), 20 mM 4-(2-hydroxyethyl)-1-piperazineethanesulfonic acid (HEPES) pH 7.4, and antibiotic/antimycotic (#15240062, Fisher Scientific, Waltham, MA, USA). Cells were incubated in a humidified environment with 5% CO_2_ at 37 °C. For most experiments, expression constructs were transfected into cells using the Amaxa^TM^ 4D-Nucleofector (10 μg plasmid cDNA; Lonza, Basel, Switzerland) or JetPrime (2 μg plasmid cDNA; Polyplus Transfection, Illkirch, France). For live-cell imaging experiments, cell nuclei were pressure injected with plasmid cDNAs (5–20 μg/mL, details below) prepared in HKCl (10 mM HEPES, 140 mM KCl, pH 7.4) using back-loaded, glass capillary needles (Flaming/Brown micropipette puller, Sutter Instrument, Novado, CA, USA) and a micromanipulator (MMO-202ND, Narishige, Greenville, NY, USA) mounted on a Nikon TS100 inverted microscope.

### 4.2. Expression Constructs

KIF1A-NR, KIF1B-NR1, KIF1B-NR2, and KIF18-NR cDNAs were synthesized by RT-PCR (SupersScript III First Strand Synthesis system by ThermoFisher, Waltham, MA, USA), amplified by PCR from human Caco_2_ cells or MCF7 breast cancer cells and cloned into Gateway^TM^ expression vectors (Invitrogen) according to the manufacturer instructions. The KIF17-NR (called KIF17-Tail in prior studies) and ERR1 constructs were described previously [14,52]. All primers used to generate additional expression constructs include the Gateway AttR1 and AttR2 cassettes for recombination. The sequences for AttR1 and AttR2 are found in the Gateway cloning manual (Invitrogen). Gene-specific primers with AttR1 and AttR2 cassettes are:

- KIF1A (NR1+NR2): forward, 5′-GAC ATG AAG CAG GAG ATG GAG and reverse, 5′-CAG GAG GTG GAG AAG ACT AGG.
- KIF1B (NR1+NR2): forward, 5′-CGC TCA GGA AAC CGT ATC ATC ATG and reverse, 5′-GTA GAC TCT AGG AGC AAC TCT CTG.
- KIF1B-NR1: forward, 5′-CCT GTG GAC TGG ACA TTT GCC and reverse, 5′-AGA CCA ATA GTG TGT TGC TCC.
- KIF1B-NR2: forward, 5′-GAT CTC TTC AGT GAC GGG and reverse, 5′-CAA GGA CAG ACC AGA ACG.
- KIF18: forward, 5′-GCT TGT CTT CAG GAA CAG CAA CAC AGG and reverse, 5′-GGA CCA GTT CAG CCT ATT CCT TGT TGC.

ERRE-Luc reporter was provided by JM Vanacker (University of Lyon, France). 3X-ERE-Luc was purchased from Addgene (Watertown, MA, USA; Plasmid 11354). KIF18A plasmids [53] used to amplify selected regions of the protein were provided by Claire Walczak (Department of Biology, Indiana University, Bloomington, IN, USA). 

All constructs were verified by sequencing prior to use in experiments. 

### 4.3. Co-Immunoprecipitation and Immunoblotting

HEK293T cells were transfected with indicated constructs in preparation for coimmunoprecipitation experiments. One day post transfection, cells were lysed in ice-cold lysis buffer (50 mM HEPES, pH 7.4, containing 150 mM NaCl, 1.5 mM MgCl_2_, 0.5 mM CaCl_2_, 10% (*v*/*v*) glycerol, 1% (*v*/*v*) Triton-X100, 1 mM phenylmethylsulfonyl fluoride (PMSF), and 0.5 mg/mL each of leupeptin, bestatin, and pepstatin) with rocking for 30 min at 4 °C. Lysates were incubated overnight at 4 °C with 4–6 μg rabbit anti-GFP antibody (Novus Biologicals, NB 600-303) or mouse anti-myc antibody (#M4439, Sigma-Aldrich, St. Louis, MO, USA) followed by immunoprecipitation with Protein-G sepharose beads (GE Healthcare, Chicago, IL, USA). Other primary antibodies used for immunoblotting include mouse anti-GFP (#1814460; Roche, Basel, Switzerland) and rabbit anti-myc (#2276S; Cell Signaling Technology, Danvers, MA, USA).

### 4.4. Luciferase Reporter Assays

Cells were transfected with indicated constructs. Twenty-four hours after transfection, cells were washed with PBS and lysed using Glo-lysis buffer (# E2661, Promega, Madison, WI, USA) for 5 min. Lysates were collected and mixed 1:1 with BrightGlo Luciferase assay reagent (#J3081, Promega) and luminescence was measured using a luminometer (SpectraMax i3x; Molecular Devices, San Jose, CA, USA). Results were corrected for background luminescence and plotted using GraphPad Prism 5. Statistical significance, *p* < 0.05, was determined using Bonferroni analysis.

### 4.5. Time-Lapse Imaging and Analysis

For live imaging of ERR1, cells were seeded onto heat-sterilized 25 mm round coverslips 1–2 days prior to cDNA microinjection. Cells were co-injected with plasmids encoding ERR1-GFP (20 μg/mL) and either mCh-KIF-NR (40 μg/mL) or mCh-empty vector control (40 μg/mL). After injections, cells were maintained in the incubator at 5% CO_2_, 37 °C for 90 min. to allow for protein synthesis. Coverslips were then transferred to recording media (Hanks balanced salt solution containing 1% FBS, 4.5 g/L glucose, essential and nonessential amino acids, 20 mM HEPES pH 7.4) and mounted in a Sykes-Moore chamber (Bellco Glass, Vineland, NJ, USA). The chamber was placed in a temperature-controlled microincubator (PDMI-2, Harvard Apparatus) on a TE2000 inverted microscope running Elements^TM^ acquisition and analysis package (NIS-Elements AR version 4.40.00; Nikon Inc., Mellville, NY, USA) and equipped with a Neo 5.5 cMOS camera (6.5 μm pixel, Andor Technologies). For MT depolymerization experiments, cells were treated with 33 μM nocodazole (NZ; #M1404 Sigma-Aldrich) immediately following cDNA injection and maintained in NZ for the duration of the experiment. Time-lapse images were acquired at 10 min intervals for 3 h using a 20× (NA 0.75) plan apochromat objective. For image analysis, background fluorescence was subtracted and regions of interest (ROIs) were identified using morphometric thresholding around individual cells and their corresponding nuclei. Integrated florescence intensities were measured for nuclear ROIs (N) and total cell area ROIs (T), and expressed as a ratio, N/T fluorescence per cell over time. Average N/T fluorescence per cell at the beginning and end of the time-lapse were plotted using Graphpad Prism 5. 

### 4.6. qRT-PCR

qRT-PCR was performed using SyBr green on a QuantStudio 7 Flex Real-time qPCR system (Applied Biosystems, Waltham, MA, USA) using standard methods. Total RNA and cDNA were obtained from stable cell lines using RNAeasy RNA extraction kit (#74104; Qiagen) and cDNA generated using standard methods. Tbp1 was used as a control. All primers were obtained from Azenta/Genewiz. The following primers were used:

- Tbp1: forward, 5′–TGT ATC CAC AGT GAA TCT TGG TTG and reverse, 5′–GGT TCG TGG CTC TCT TAT CCT C.
- 14-3-3: forward, 5′–GCATGAAGTCTGTAACTGAGCA and reverse, 5′–GCACCTTCCGTCTTTTGTTC.
- Claudin-4: forward, 5′–CCATATAACTGCTCAACCTGTCC and reverse, 5′–AGATAAAGCCAGTCCTGATGC.
- ERR1: forward, 5′–TCTCCGCTTGGTGATCTCA and reverse, 5′–CTATGGTGTGGCATCCTGTG.
- HIF1A: forward, 5′–CAACCCAGACATATCCACCTC and reverse, 5′–CTCTGATCATCTGACCAAAACTCA.
- HIF2: forward, 5′–CTTTGCGAGCATCCGGTA and reverse, 5′–AGCCTATGAATTCTACCATGCG.
- Osteopontin: forward, 5′–CCCCACAGTAGACACATATGATG and reverse, 5′–TTCAACTCCTCGCTTTCCAT.
- PGC1A: forward, 5′–GTC CTT TTC TCG ACA CAG GT and reverse, 5′–GTC TGT AGT GGC TTG ACT CAT AG.

## Figures and Tables

**Table 1:** Human kinesin family motors. The amino acid sequences of all human kinesin family motors, organized by subfamily, were scanned for the presence of LxxLL motifs (red text). Kinesins with LxxLL motifs in only the microtubule-binding motor domain, neck-coil region, coiled-coil domains, and N-terminal extension regions were eliminated. LxxLL motifs in the stalk and tail (defined here as regions beyond the last coiled-coil) domains were then assessed for the presence of a monopartite nuclear localization signal. Kinesins meeting these criteria for analysis are highlighted in boxes.

Scan Kinesins for LxxLL Motif --> Define Kinesin Domain Containing LxxLL --> Scan for Monopartite, Classical NLS --> Include/Exclude									
**Kinesin Family**	**LxxLL (+/− 6 amino acids)**	**Position in Sequence**	**Kinesin Domain**	**NLS**	**Kinesin Family**	**LxxLL (+/− 6 amino acids)**	**Position in Sequence**	**Kinesin Domain**	**NLS**
**Kinesin-1 family**					**Kinesin-6 family**				
KIF5A	None	N/A	N/A	+	KIF20A	LHPTPDLKPLLSNEVIW	201–205	motor	−
KIF5B	None	N/A	N/A	−		EIYNELLYDLLEPPSQQ	313–317	motor	
KIF5C	QEREMKLEKLLLLNDKR	767–771	coiled-coil	−		DEKIEELEALLQEARQQ	651–655	coiled-coil	
						PGKKPFLRNLLPRTPTC	850–854	tail	
**Kinesin-2 family**					KIF20B	None	N/A	N/A	−
					KIF23	None	N/A	N/A	+
KIF3A	PYRNSKLTRLLQDSLGG	302–306	motor	+					
KIF3B	PYRDSKLTRLLQDSLGG	297–301	motor	+	**Kinesin-7 family**				
KIF3C	PYRDSKLTRLLQDSLGG	322–326	motor	−					
**KIF17**	PYRDSKLTRLLQDSLGG	287–301	motor	+	KIF10/CENPE	AMEKDQLAQLLEEKDLL	376–380	coiled-coil	+
	QMSPSSLSALLSRQVPP	368–382	coiled-coil						
	EPQEVPLQGLLGLQDPF	601–615	stalk		**Kinesin-8 family**				
	ERDSMLLQQLLEQVQPL	852–866	tail						
					KIF18A	PYRNSKLTRLLKDSLGG	312–316	motor	+
**Kinesin-3 family**						RQEYLKLEMLLKENELK	443–447	coiled-coil	
						RQTEAVLNALLPTLRKQ	573–577	stalk	
**KIF1A**	PYRDSVLTWLLRENLGG	311–315	motor	+	KIF18B	PYRDSKLTRLLKDSLGG	308–312	motor	+
	KDEVTRLRDLLYAQGLG	379–383	motor		KIF19	NYRDSKLTRLLKDSLGG	303–307	motor	+
	WYQFTSLRDLLWGNAIF	722–726	stalk						
	GRAFVYLSNLLYPVPLV	857–861	tail		**Kinesin-9 family**				
**KIF1B**	PYRDSVLTWLLRENLGG	311–315	motor	+					
	KEEVTRLKDLLRAQGLG	379–383	neck-coil or coiled-coil		KIF6	HHCFHHLKKLLNDKKIL	428–432	stalk	−
	SHQFTSLRDLLWGNAVY	762–766	stalk		KIF9	EIYNESLFDLLSTLPYV	149–153	motor	−
	GRAFVYLSNLLYPVPLI	980–984	tail						
KIF1C	PYRDSVLTWLLKENLGG	305–309	motor	+	**Kinesin-10 family**				
	QEEVARLRELLMAQGLS	373–377	motor						
KIF13A	PYRDSVLTWLLKDNLGG	309–313	motor	+	KIF22	GVIPRALMDLLQLTREE	151–155	motor	+
	LNADPALNELLVYYLKD	458–462	stalk			PYRDSKLTRLLQDSLGG	325–329	motor	
KIF13B	PYRDSVLTWLLKDSLGG	310–314	motor	+		SMDPAMLERLLSLDRLL	440–444	stalk	
	LNADPALNELLVYYLKE	459–463	stalk			LERLLSLDRLLASQGSQ	446–450	stalk	
KIF14	PYRESVLTWLLKESLGG	658–662	motor	−					
	VLMKHWLSDLLPCTNIA	1325–1329	stalk		**Kinesin-11 family**				
KIF16A	PYRDSVLTWLLKDSLGG	341–345	motor	+					
	REEIERLKALLLSFELR	409–413	coiled-coil		KIF26A	TLRDPCLSALLLDKLPA	100–104	N-terminal region	+
	AASRLGLSPLLWKERRA	617–621	stalk			CGRDQSLRDLLAEVAPG	530–534	motor	
	AYLKNNLPVLLQNQNSK	1798–1802	stalk			PYRDHRLTMLLRESLAT	681–685	motor	
	HNLSLHLSQLLHSTSEL	3684–3688	stalk		KIF26B	WGKEENLRDLLSEVATG	607–611	motor	+
KIF16B	PYRDSVLTWLLKDSLGG	315–319	motor	−		PYKESKLAMLLRESLGN	757–761	motor	
	RAEIARLKTLLAQGNQI	383–387	coiled-coil						
	FHIENKLKDLLAEKEKF	669–673	coiled-coil		**Kinesin-12 family**				
	QYKERQLQYLLQNHLPT	909–913	stalk						
	YLLQNHLPTLLEEKQRA	917–921	stalk		KIF12	FGSLEALMELLQTGLSR	204–208	motor	−
KIF28	PYRDSVLTKLLQSALGG	312–316	motor	−		PFRDSKLTKLLADSLGG	317–321	motor	
					KIF15	CYRDSKLTFLLRDSLGG	320–324	motor	−
**Kinesin-4 family**						QKANLNLENLLEATKAC	630–634	coiled-coil	
						KSDLNNLMELLEAEKER	902–906	coiled-coil	
KIF4A	PYRDSKLTRLLQDSLGG	293–297	motor	+		PHFQTHLAKLLETQEQE	1152–1156	coiled-coil	
	KQQVQQLQVLLLQAHGG	361–365	motor						
	EEAKRHLNDLLEDRKIL	764–768	coiled-coil		**Kinesin-13 family**				
KIF4B	PYRDSKLTRLLQDSLGG	293–297	motor	+					
	KQQVQQLQVLLLQAHGGT	361–365	coiled-coil		KIF2A	None	N/A	N/A	−
	EEAKRHLNDLLEDRKIL	764–768	coiled-coil		KIF2B	IEEVETLPTLLGKDTTI	601–605	C-terminal domain	+
KIF7	None	N/A	N/A	+	KIF2C	AAINPELLQLLPLHPKD	68–72	N-terminal domain	−
KIF21A	PYRDSKLTRLLQDSLGG	328–332	motor	+		EIYNGKLFDLLNKKAKL	409–413	motor	
	AELNPELDALLGHALQD	1097–1101	tail		KIF24	EIYCGQLYDLLNRRKRL	367–371	motor	−
KIF21B	PYRDSKLTRLLQDSLGG	327–331	motor	+					
KIF27	EVYKEDLRDLLELETSM	143–147	motor	+	**Kinesin-14 family**				
					KIFC1	PYRNSKLTYLLQNSLGG	620–624	motor	+
**Kinesin-5 family**					KIFC2	LTVTSQLLALLAWLRSP	124–128	N terminal domain	−
						PFRDSQLTRLLQPALGP	687–691	motor	
KIF11	IYNEELFDLLNPSSDV	168–172	motor	−	KIFC3	EIYNEVLRDLLGKEPQE	582–586	motor	+
	NEREQELHNLLEVVSQC	844–848	tail			PFRNSKLTYLLQDSLSG	725–729	motor	
					KIF25	GDVCPLLTSLLDGYNVC	50–54	motor	−
						AALAGVLGALLEHRGHA	303–307	motor	
						PYRNSRLTHLLQDCLGG	320–324	motor	

## Data Availability

Not applicable.
